# The Present and Future of Regional Anaesthesia in Türkiye

**DOI:** 10.4274/TJAR.2025.252257

**Published:** 2025-10-14

**Authors:** Serkan Tulgar, Alper Kılıçaslan, Özlem Selvi Can, Zekeriyya Alanoğlu

**Affiliations:** 1 Bahçeşehir University Faculty of Medicine, Göztepe Medical Park Hospital, Department of Anesthesiology and Reanimation, İstanbul, Türkiye; 2 Necmettin Erbakan University Faculty of Medicine, Department of Anesthesiology and Reanimation, Konya, Türkiye; 3 Ankara University Faculty of Medicine, Department of Anesthesiology and Reanimation, Ankara, Türkiye; 4 Washington University in St. Louis, School of Medicine, Department of Anesthesiology St. Louis, United States

## Editorial

In examining the current position of regional anaesthesia in Türkiye, this comprehensive study provides insights on several distinct levels: from the practitioners’ perspective, it delineates the preferences of colleagues across different age groups and the distribution of these choices within diverse institutions. From the vantage point of practice, it highlights the breadth of techniques and the nuances of case-based selection, and finally, from the patients’ perspective, the study brings into focus the reasons for refusal, age-related patterns, and the spectrum of complications. This editorial draws on the paper by Kanat et al.1 published in this edition of the Turkish Journal of Anaesthesiology and Reanimation, to present these dynamics in greater depth and to contextualize them within the broader evolution of regional anaesthesia practice. In light of these findings, it is our endeavor to elevate the discourse on regional anaesthesia to a higher plane.

In the last decade, regional anaesthesia has undergone a global transition from neuraxial techniques to peripheral approaches, moving beyond plexus blocks toward the interfascial blockade of  the most distal terminal branches. In Türkiye, this trend has gained exceptional momentum, driven by Turkish scholars whose unwavering focus on regional anaesthesia has propelled the field with remarkable force. Across every tier of our healthcare system, anesthesiologists of all generations have gained hands-on experience with fascial plane blocks, and many have subsequently contributed their insights to the academic reservoir, further enriching the collective knowledge of the discipline. It is, however, apparent that regional anaesthesia in pediatric patients and the use of catheters in regional anaesthesia remain underutilized. We particularly wish to encourage our colleagues to advance practice and research in these areas, always with the proviso that appropriate safeguards and a secure clinical environment are in place.

While reporting techniques and studies in regional anaesthesia that capture the current state of the art and at times even surpass the average, it is crucial for Turkish anesthesiologists, whether publishing in our journal or in other esteemed international outlets, to exercise meticulous attention to prospective clinical trial registration and proper sample size calculation. To minimize practices that may undermine scientific credibility and to avoid irreparable methodological errors, we strongly encourage colleagues to seek mentorship from seasoned academics who have already advanced along this path. For practicing anesthesiologists who may not be directly engaged in research, the significance of their daily clinical choices should not be underestimated. The cumulative expertise gained in routine practice, whether in the breadth of block applications or in the vigilance toward patient safety, constitutes the very foundation upon which academic progress is built. We therefore encourage all colleagues, regardless of their academic involvement, to recognize the value of their practice and to share their insights whenever possible.

In this way, regional anaesthesia in Türkiye not only reflects global currents but also stands poised to shape its future trajectory.
